# Effects of high intake of cod or salmon on gut microbiota profile, faecal output and serum concentrations of lipids and bile acids in overweight adults: a randomised clinical trial

**DOI:** 10.1007/s00394-020-02417-8

**Published:** 2020-10-27

**Authors:** Marianne Bratlie, Ingrid V. Hagen, Anita Helland, Friedemann Erchinger, Øivind Midttun, Per Magne Ueland, Grethe Rosenlund, Harald Sveier, Gunnar Mellgren, Trygve Hausken, Oddrun Anita Gudbrandsen

**Affiliations:** 1 Dietary Protein Research Group, Department of Clinical Medicine, Haukeland University Hospital, University of Bergen, 5021 Bergen, Norway; 2 Department of Clinical Medicine, University of Bergen, Bergen, Norway; 3 Bevital AS, Jonas Lies veg 87, 5021 Bergen, Norway; 4 Skretting Aquaculture Research Centre AS, P.O. Box 48, 4001 Stavanger, Norway; 5 Lerøy Seafood Group ASA, P.O. Box 7600, 5020 Bergen, Norway; 6 Mohn Nutrition Research Laboratory, Department of Clinical Science, Haukeland University Hospital, University of Bergen, 5021 Bergen, Norway; 7 Hormone Laboratory, Haukeland University Hospital, 5021 Bergen, Norway; 8 Section of Gastroenterology, Department of Medicine, Haukeland University Hospital, Bergen, Norway

**Keywords:** Cod, Salmon, Gut microbiota, Faeces, Lipids

## Abstract

**Purpose:**

To explore whether high intake of cod or salmon would affect gut microbiota profile, faecal output and serum concentrations of lipids and bile acids.

**Methods:**

Seventy-six adults with overweight/obesity with no reported gastrointestinal disease were randomly assigned to consume 750 g/week of either cod or salmon, or to avoid fish intake (Control group) for 8 weeks. Fifteen participants from each group were randomly selected for 72 h faeces collection at baseline and end point for gut microbiota profile analyses using 54 bacterial DNA probes. Food intake was registered, and fasting serum and morning urine were collected at baseline and end point.

**Results:**

Sixty-five participants were included in serum and urine analyses, and gut microbiota profile was analysed for 33 participants. Principal component analysis of gut microbiota showed an almost complete separation of the Salmon group from the Control group, with lower counts for bacteria in the *Bacteroidetes* phylum and the *Clostridiales* order of the *Firmicutes* phyla, and higher counts for bacteria in the *Selenomonadales* order of the *Firmicutes* phylum. The Cod group showed greater similarity to the Salmon group than to the Control group. Intake of fibres, proteins, fats and carbohydrates, faecal daily mass and output of fat, cholesterol and total bile acids, and serum concentrations of cholesterol, triacylglycerols, non-esterified fatty acids and total bile acids were not altered in the experimental groups.

**Conclusion:**

A high intake of cod or salmon fillet modulated gut microbiota but did not affect faecal output or serum concentrations of lipids and total bile acids.

**Clinical trial registration:**

This trial was registered at clinicaltrials.gov as NCT02350595.

**Electronic supplementary material:**

The online version of this article (10.1007/s00394-020-02417-8) contains supplementary material, which is available to authorized users.

## Introduction

Gut microbiota may affect human nutrition and health by actively contributing to food digestion, energy harvesting and production of metabolic active compounds including short chain fatty acids and bile acids, but a definition of healthy human gut microbiome is still lacking [1]. The breakdown of proteins from the diet, followed by absorption of small peptides or free amino acids in the small intestine, is an efficient process in healthy adults; however even easily digestible proteins such as egg proteins escape digestion and absorption in the small intestine and may serve as substrate for microbial energy metabolism in the colon to produce mainly short chain fatty acids [2, 3]. Also dietary fibres, some starch and very small quantities of simple sugars enter the colon for fermentation to short chain fatty acids by bacteria [2].

The short chain fatty acids acetate, propionate and butyrate are generally accepted to be beneficial for the gut function and morphology [4], and improve glycaemic control and reduce appetite in addition to serve as an energy source for the host [5]. Gut microbiota also plays important roles in the microbial deconjugation and dehydroxylation of bile acids and in the enterohepatic circulation of bile acids, and may directly regulate circulating cholesterol concentration [6, 7]. Glycine or taurine conjugated bile acids produced from cholesterol in the liver is deconjugated to secondary bile salts by bile salt hydrolases, found in all major bacterial divisions, before reabsorption as a part of the enterohepatic cycle [6, 7]. Thus, changes in diets can be expected to affect the gut microbiota and through changes in physiological processes this may affect health and development of diseases.

Clinical studies have shown that fish intake or fish protein supplementation is associated with increased HDL-cholesterol and/or lower LDL-cholesterol concentrations [8–13]. Lower circulating cholesterol concentration is also found in rats fed fish or fish proteins [14–20], and it has been suggested that this may in part be regulated through a higher faecal bile acid excretion [14, 19–21]. A reduction in circulating cholesterol concentration through increased faecal bile acid output may therefore be associated with changes in gut microbiota composition.

The information on the effect of diets on gut microbiota composition and function in humans is scarce. Short-term effects on the microbial community structure have been reported in healthy adults after intake of plant- or animal-based diets, or high-fat/low-fibre or low-fat/high-fibre diets [22, 23], and non-digestible carbohydrates (dietary fibres) beneficially affect gut function and affects gut microbiota [24]. The effects of intake of protein-rich foods such as fish on gut microbiota composition and function are still little investigated. A 4-week crossover study with 2235 g of lean seafood per week showed only marginal effects on gut microbiome composition [25], and also supplementation with an n-3 PUFA preparation with high EPA content (2–10 g/day) for 12 weeks in healthy humans had little effect on intestinal bacteria [26]. When fed to rats, proteins from fish affected the composition of gut bacteria in the caecum differently from proteins from beef, pork, casein or soy, resulting in higher abundance of *Firmicutes* and less *Bacteroidetes* [27]. Fish may also affect gut microbiota due to its content of trimethylamine N-oxide (TMAO), found in various fish species including Atlantic salmon and Atlantic cod [28]. TMAO may reduce bile acid production from cholesterol in liver [29], and evidence suggests that changes in bile acid pool size can affect the gut microbiome community structure [30].

In a previous paper, we showed that a high intake of Atlantic salmon (750 g/week) for 8 weeks improved postprandial glucose regulation in study participants with overweight/obesity, whereas high intake of Atlantic cod (750 g/week) did not affect glucose regulation in this study setting [31]. The TMAO content is higher in cod compared to salmon [28], and we recently demonstrated that high cod intake, but not high salmon intake, markedly increased the TMAO concentration in serum and urine of this study population as a result of high TMAO intake from cod fillet [28]. In the present study, we aimed to further explore the biological materials from this randomised clinical trial to investigate if a high intake of cod or salmon would affect gut microbiota composition, faecal output and serum concentrations of lipids and bile acids. Little is known about the effects of high intake of cod or salmon on gut microbiota composition, and whether this could be linked to lipid homeostasis. Our hypothesis was that high intake of cod or salmon would affect gut microbiota and faecal output of lipids and bile acids, and thus improve circulating lipid status in a group of adults with overweight/obesity.

## Methods

### Participants, study setting and ethics

The study design, the description of study participants, the study setting and the protocol for study visits have been published in detail previously [31]. In brief, the study population consisted of adults with overweight or obesity, and all participants were of Norwegian ethnic origin (Caucasian) living in the Bergen area in South-Western Norway. Inclusion criteria were: BMI ≥ 27 kg/m^2^, fasting blood glucose ≤ 7.0 mmol/l and age 18–69 years. Exclusion criteria were high habitual fish/seafood intake (> 500 g/week), pregnancy, incompatibility with fish consumption (allergies, intolerance and/or dislike), diagnosed diabetes mellitus, heart disease or gastrointestinal diseases, use of medications affecting lipid metabolism or glucose homoeostasis, use of anti-inflammatory medications, use of supplements containing n-3 PUFAs, intentional weight loss and large fluctuation in body weight (> 3 kg) over the previous 2 months. Participants were interviewed about their fish/seafood intake before they were included in the study, and those with a regular fish intake > 1 fish dinner per week were instructed to avoid eating fish for 4 weeks before the baseline visit. Very few participants (< 10%) had an intake above 1 fish dinner per week before enrolment to the study and went through a 4**-**week fish-free period, and those with a habitual fish/seafood intake > 500 g/week were not included in the study.

The study was designed as a randomised, controlled intervention study with a parallel group design, with three intervention arms: Atlantic cod (wild-caught *Gadus morhua*) in weekly doses of 750 g, Atlantic salmon (farmed *Salmo salar*) in weekly doses of 750 g, and no-fish intake as the Control group. The intervention period was 8 weeks. In total, 76 participants were included in the study and were randomly assigned to the Cod group (*N* = 27), the Salmon group (*N* = 27) or the Control group (*N* = 22). The participants were randomised into the different groups by the project manager by drawing lots, and the participants were informed about their group allocation during the baseline visit. All examinations were conducted at the Clinical Research Unit at the Haukeland University Hospital, Bergen, Norway. To enhance compliance, the participants were contacted by phone approximately 1 week prior to baseline and end point visits, during which they were informed of the schedule and procedures for the following visit. Also, a text message was sent 1–3 days before the 8-week visit, as a reminder of how to prepare for the upcoming visit. For any inquires during the trial period, members of the research group could be reached by email or telephone. Compliance was monitored through interviews; after 1, 4 and 8 weeks intervention, the participants in the fish-eating groups were asked how many dinners with cod/salmon they had not eaten since the last contact, instead of asking how well they had complied, to lower the bar for reporting missing intake. Participants in the Control group were interviewed about their intake of fish/seafood after 1, 4 and 8 weeks. Noncompliance was defined as not following the protocol with regard to fish intake (omitting more than 3 fish dinners in the fish-eating groups), other dietary changes or use of prescription medicine not compatible with the inclusion criteria, or changes in physical activity. As reward for completing the study, participants were offered a dietary consultation with a student dietician and all results from blood analyses.

The study was conducted according to the guidelines laid down in the Declaration of Helsinki, and all procedures were approved by the Regional Committee for Medical and Health Research Ethics of Western Norway (REC no.: 2011/572). Written informed consent was obtained from all subjects.

Health professionals performing blood sampling, and personnel conducting the laboratory analyses, were all blinded to participants’ identity and group allocation. All data were analysed anonymously. This trial was registered at clinicaltrials.gov as NCT02350595.

### Interventions

Cod and salmon fillets were provided to the participants as frozen skin- and boneless fillet portions (mean weight with standard deviation; 150 (SD 10) g; Lerøy Seafood Group ASA), and pallets of fish were chosen at random from Lerøy’s warehouse in Bergen, Norway. The cod and salmon fillets were supplied free of charge to the participants, and were distributed at the baseline visit or at any time during the study period, if preferred. Participants in the Cod group were instructed to eat five dinners per week containing 150 g of cod fillet, and participants in the Salmon group were instructed to eat five dinners per week containing 150 g of salmon fillet. The participants in the fish-eating groups were instructed not to exceed a total amount of 750 g of fish/week, not to consume any other types of fish or seafood during the study period, and to otherwise maintain their normal eating habits throughout the study period apart from eating the mandatory amount of 750 g fish/week. The Control group was also instructed to continue their normal eating habits, except to avoid fish and seafood intake. Participants in both fish intervention groups received a booklet with recipes for inspiration and to help them to increase the variation of their meals, as previously described [13]. The participants in the Control group did not receive any recipes, to avoid them being inspired to change their dietary habits during the study period.

Subjects in all groups were instructed not to change their physical activity level during the intervention period. The participants’ dietary intake and habitual lifestyle were recorded at the baseline and the end point visits, using food record charts and a questionnaire for reporting physical activity. The participants were asked about the types of physical activity they engaged in, such as whether they worked out in a gym, were members of sports clubs or whether they worked out individually, the type of physical activity (e.g. hiking, running, biking) and the number of hours of light physical activity (not sweaty/not breathless) or hard physical activity (with sweat/breathless). The participants completed the questionnaire at the baseline and end point visits. The weekly number of hours and the intensities of the physical activities were coded as continuous variables. Reported energy and macronutrient intake and physical activity were similar between the groups at baseline and did not change within the groups during the study period [31].

### Protocol for study visits

The total study period was 8 weeks, with baseline visits between August 22, 2011 and September 19, 2011. Examinations were conducted in the morning after an overnight fast. The participants were instructed not to eat or drink anything except water, and not to use substances containing nicotine after 22.00 h the previous day, and to avoid physical exercise and alcohol for 24 h before each visit.

Body height was measured at the baseline, using a wall-mounted stadiometer (Seca 222; Seca). Body weight and body composition were measured in a fasted state using a bioelectrical impedance analysis device (InBody 720; Biospace Co. Ltd) at the baseline and the end point visits.

Fasting blood samples and morning urine were collected at baseline and end point. Blood was drawn into BD Vacutainer SST II Advance gel tubes (Becton, Dickinson and Company) for isolation of serum and in Vacuette K2EDTA tubes (Greiner Bio-one, Austria) for collection of whole blood. The staff complied with a strict protocol for pre-analytical sample handling to ensure high sample quality. Serum, whole blood and urine were aliquoted and frozen at − 80 °C until analyses.

### Estimation of dietary intakes

Participants completed dietary records of the five preceding days before the baseline visit and the 8-week visit, including at least 1 weekend day. The intakes of non-digestible dietary fibre, starch, total fat, cholesterol and protein were calculated from the participants’ dietary records using the ‘Mat på Data 5.1’ software [32], which contains information on the nutrient contents in food items sold in Norway. In this database, dietary fibres are defined as non-digestible carbohydrates, and the collective term carbohydrate is used for available carbohydrates, i.e. including sugars and starch but not dietary fibres. Food records were checked for completeness at both study visits.

### Faeces collection and analysis

Fifteen subjects from each experimental group were selected by the project manager (by drawing lots) to do a 72-h faeces collection at baseline and end point, and the participants were informed about this during the baseline visit. Participants were interviewed about their opportunity to store faeces in their home freezer before receiving equipment for collection of faeces. The participants received detailed written and verbal instructions on how to conduct a 72-h faeces collection and were provided with appropriate plastic containers with tight lids (1 l) and a holder to support the containers in the toilet. Faeces were collected on three subsequent days, and participants in the fish groups were instructed to complete the baseline faeces collection before they started consuming cod or salmon.

The participants were instructed to store the samples immediately after each bowel movement in a new container in their own freezer, and they could deliver the faeces samples to our laboratory at any time throughout the intervention period. The faecal samples were stored at − 20 °C until further processing. The faeces were weighed in day by day and the total faeces from 72 h collection for each participant at each time point were pooled, mixed with twice the amount of water, homogenised with a T 50 basic ULTRA-TURRAX® (IKA® Werke, Staufen, Germany) and stored at − 20 °C. Aliquots of faecal samples were weighed before and after freeze drying and the dry mass content of the stool samples was calculated.

Gut microbiota profile analysis was performed using the GA-map® Dysbiosis Test (Genetic Analysis AS, Oslo, Norway) by algorithmically assessing faecal bacterial abundance [33]. Briefly, the test is based on mechanical and chemical bacterial cell disruption and automated total bacterial genomic DNA extraction using magnetic beads, followed by PCR amplification of the 16S rRNA gene. 54 DNA bacterial markers targeting more than 300 bacteria based on their 16S rRNA sequence in seven variable regions (V3–V9) were analysed; 28 bacteria probes are species specific, 18 detect bacteria at genus level and 8 probes detect bacteria at higher taxonomic levels. Probes are listed in Supplemental Table 1. Probe labelling is by single nucleotide extension and hybridisation to complementary probes coupled to magnetic beads, and signal detection by using BioCode 1000A 128-Plex Analyzer (Applied Bio-Code, Santa Fe Springs, CA, USA).

Cholesterol was measured in lipid extracts from faeces as previously described [34, 35] using the Cholesterol Gen.2 kit for Cobas c111 from Roche Diagnostics GmbH (Mannheim, Germany). Total bile acids (3α-hydroxy bile acids) concentration was measured in freeze-dried faeces after solid phase extraction with Chromabond C18 ec (3 ml/200 mg, Macherey–Nagel, Düren, Germany) as described by Suckling et al. [36] and quantification using the enzymatic bile acid assay from Diazyme Laboratories, Inc. on the Cobas c111 system (Roche).

Faecal fatty acids were quantified using a modified van de Kamer method, described in detail elsewhere [37, 38]. In brief, acylglycerols and cholesterol esters were hydrolysed by boiling homogenised faecal samples with KOH and ethanol, before acidifying with HCl. Free fatty acids were extracted from the mixture using petroleum ether, and the molar amount of fatty acids was determined by titration against 0.1 N NaOH using thymol blue as an indicator.

### Analyses of serum, whole blood and urine samples

Analyses of total cholesterol, HDL-cholesterol, LDL-cholesterol, triacylglycerol, total bile acids, glucose, C-reactive protein (CRP) and alanine transaminase (ALT) in blood serum were performed by routine methods at the Laboratory of Clinical Biochemistry at Haukeland University Hospital. Serum NEFA was analysed with the Cobas c111 system (Roche Diagnostics GmbH, Marburg, Germany) using the NEFA FS kit (DiaSys; Diagnostic Systems GmbH). Urine creatinine and HbA1c in whole blood were analysed on the Cobas c 111 system using the CREP2 (Creatinine plus ver.2) kit and the A1C-3 kit with A1CD2 haemolysing reagent (Roche Diagnostics GmbH) for Cobas c111. Trimethylamine N-oxide (TMAO) and 1-methylhistidine (1-MeHis, π-methylhistidine) were measured in serum and urine at Bevital AS (Bergen, Norway, https://www.bevit al.no) using liquid chromatography combined with tandem mass spectrometry, as previously described [39] by adding ion pairs for the analytes and isotope-labelled internal standards to the existing assay.

### Outcome measurements

The primary outcome of the present study was the gut microbiota profile after a weekly intake of 750 g fillet from either cod or salmon, or a fish-free diet for 8 weeks. Secondary outcomes were changes in faecal output and serum concentrations of lipids and bile acids after cod or salmon intake, and possible correlations between markers of cod and salmon intake and gut microbiota.

### Sample size estimation

The sample size calculation for this trial was originally conducted with the aim of investigating the effects of high intake of cod or salmon on postprandial glucose regulation after a standardised breakfast in participants with overweight or obesity [31]. We estimated that it was necessary to include 76 participants divided into three groups to ensure that 20 participants in each group completed the trial with satisfactory compliance, with a power of 80% and α of 0.05, and of these, 65 participants were included in statistical analyses [31]. In the present study, we wanted to further explore the biological materials from this randomised clinical trial to investigate the effects of a high intake of cod or salmon on gut microbiota composition, faecal output of lipids and bile acids, and circulating lipids. The primary aim of the present study was to investigate the effects of high intake of cod or salmon on gut microbiota. Forty-five participants (15 participants in each experimental group) were selected for collection of all faeces produced in 72 h at baseline and end point, and of these three participants withdrew from the study, four participants were not able to complete their collection of faeces, and one participant was excluded from analysis due to poor compliance; thus, a total of 37 sets of faeces samples were collected for gravimetric and biochemical analyses. We were able to obtain complete microbiota profiles from faeces sampled at both baseline and end point from 33 participants.

This is the first study to investigate the effects of 8 weeks of high intake (750 g/week) of cod or salmon on gut microbiota and faecal output in adults with overweight/obesity; therefore, no data were available for sample size calculation for the present study.

### Statistical analyses

Subjects who did not complete the study were excluded from the statistical analyses. For lipids and bile acids in faeces and serum, and for estimated intake of nutrients from dietary records, most data were not normally distributed according to the Shapiro–Wilk test, and nonparametric tests were used to investigate changes within groups (the Wilcoxon-signed ranks test). For these nonparametric data, the Kruskal–Wallis test was used to compare values between the three groups at baseline. Changes within the groups were compared using univariate analysis of covariance (ANCOVA) with adjustment for baseline values after log transformation. Data are expressed as medians and 25th, 75th percentiles. Categorical data were compared using the Pearson’s *χ*^2^ test. All comparisons were two-sided. Correlations between parameters were tested using two-tailed Spearman’s correlations test. Statistical analyses were conducted using SPSS Statistics 25 (SPSS, Inc., IBM Company) and *P* < 0.05 was considered statistically significant. Patterns of microbiota profiles were investigated by performing principal component analysis (PCA) on matrixes containing centred and standardised concentrations using SigmaPlot 14.0 (Systat Software, Inc.).

## Results

### Participant characteristics

**Fig. 1:**
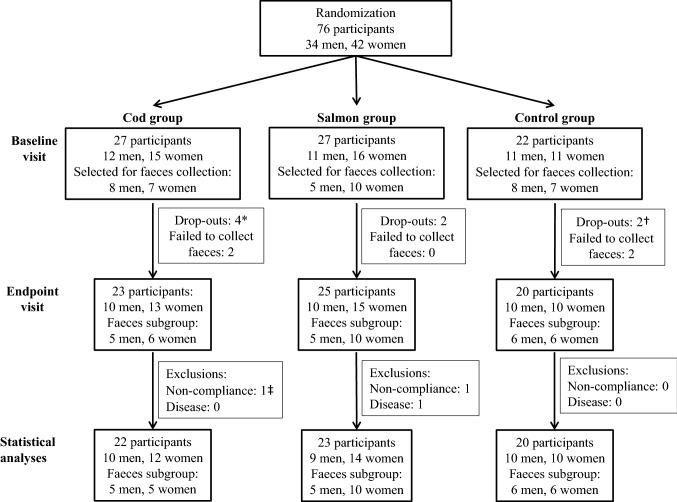
Study overview of participants. Participants not complying with the protocol were not included in the statistical analyses. Non-compliance was defined as not following the protocol in regard to fish intake (omitting more than 3 fish dinners in the fish-eating groups), other dietary changes or use of prescription medicine not compatible with the inclusion criteria, or changes in physical activity. *Of these, two participants (one man and one woman) in the Cod group were selected for faeces collection. ^†^Of these, one participant (a woman) in the Control group was selected for faeces collection. ^‡^Of these, one participant (a woman) selected for faeces collection in the Cod group was excluded from analysis

Seventy-six participants were included in the study and completed the first study visit, and 68 participants completed the trial. Two participants (one woman in the Cod group and one man in the Salmon group) were excluded from analysis because they did not comply with the protocol, and one woman in the Salmon group was excluded from statistical analysis after analyses of postprandial blood glucose revealed that she had prediabetes. In total, 65 subjects (29 men and 36 women) were included in the statistical analyses. The flow of participants in the study is presented in Fig. 1. The groups were similar at baseline in regard to sex distribution, age, BMI, percentage body fat, percentage muscle mass, reported light (not sweaty/not breathless) and hard (with sweat/breathless) physical activity, and fasting serum concentrations of glucose, CRP and ALT (Table 1), with median age 45.5 (25th, 75th percentiles 36.2, 53.2) years and median BMI 32.3 (25th, 75th percentiles 29.6, 35.7) kg/m^2^. After 8 weeks, no changes were seen in any of the groups for BMI, percentage body fat or muscle mass (data not presented). None of the participants reported using antibiotics, probiotics or prebiotics at baseline or end point, or at any time during the intervention period.

**Table 1:** Participant characteristics at baseline: overview of all participants that completed the study and were included in the statistical analysis

	Cod group (*N* = 22)		Salmon group (*N* = 23)		Control group (*N* = 20)		P^*^
Men/women	10/12		9/14		10/10		0.77
Age, years	47.2	38.0, 54.2	45.9	43.1, 52.2	39.8	31.1, 52.7	0.39
BMI, kg/m^2^	31.0	29.2, 35.9	31.9	29.9, 34.6	34.4	29.3, 36.5	0.64
Body fat, %	39.3	28.3, 42.9	39.9	30.0, 42.9	39.1	32.5, 41.0	0.99
Muscle mass, %	34.4	32.5, 41.4	33.3	31.5, 40.0	34.0	32.4, 38.5	0.85
Light physical activity, h/week	1	0, 4	2	2, 3	2	1, 3	0.35
Hard physical activity, h/week	2	0, 3	2	1, 2	2	0, 3	0.97
Glucose, mmol/l	5.2	4.9, 5.6	5.2	4.9, 5.5	5.3	4.8, 5.6	0.96
CRP, mg/l	2.0	0.78, 3.25	2.0	0.9, 3.0	2.5	2.0, 5.8	0.084
ALT, U/l	27	20, 43	26	17, 39	28	16, 36	0.56

Medians and 25th and 75th percentiles

*CRP* C-reactive protein, *ALT* alanine transaminase

^*^Groups were compared at the baseline using the Pearson’s *χ*^2^ (categorical data) or the Kruskal–Wallis test (continuous data). Glucose, CRP and ALT were measured in serum collected in a fasting state

Fifteen participants from each of the experimental groups were chosen for collection of two periods of faeces for 72 h at baseline and at the end of the intervention period. Of these 45 participants, 3 withdrew from the study (1 man and 1 woman in the Cod group, 1 woman in the Control group) and 4 men (2 in the Cod group and 2 in the Control group) were not able to complete their collection of faeces. One woman in the Cod group was excluded from analysis due to poor compliance. Table 2 presents the baseline characteristics for the 37 participants collecting faeces.

**Table 2:** Participant characteristics at baseline: overview of participants that collected faeces

	Cod group (*N* = 10)		Salmon group (*N* = 15)		Control group (*N* = 12)		P^*^
Men/women	5/5		10/5		6/6		0.58
Age, years	49.4	36.6, 54.5	46.4	43.2, 53.9	39.8	31.1, 52.7	0.28
BMI, kg/m^2^	30.4	28.8, 36.7	31.5	29.5, 34.6	33.3	29.1, 36.2	0.90
Body fat, %	37.9	28.2, 40.8	39.9	30.0, 42.2	39.1	33.0, 40.1	0.84
Muscle mass, %	34.6	32.6, 40.7	33.3	31.8, 40.0	34.0	33.2, 37.9	0.81
Light physical activity, h/week	1	1, 3	2	1, 3	2	1, 3	0.81
Hard physical activity, h/week	2	0, 4	2	1, 2	2	0, 3	0.80
Glucose, mmol/l	5.2	4.9, 5.6	5.3	5.1, 5.6	5.2	4.7, 5.8	0.75
CRP, mg/l	2.0	0.9, 4.3	2.0	0.9, 3.0	4.0	2.0, 9.8	0.23
ALT, U/l	25	20, 44	25	17, 32	23	15, 34	0.56

Medians and 25th and 75th percentiles

*CRP* C-reactive protein, *ALT* alanine transaminase

^*^Groups were compared at the baseline using the Pearson’s *χ*^2^ (categorical data) or the Kruskal–Wallis test (continuous data). Glucose, CRP and ALT were measured in serum collected in a fasting state

### Estimated dietary intakes and calculated faecal output

The food intake was registered by participants for 5 days before the baseline and end point visits. We have previously shown that there was no difference in estimated energy intake from baseline to end point within any of the groups [31]. Here, we show that the estimated median intakes of non-digestible dietary fibre, starch, total fat, protein, cholesterol and carbohydrates were not different between the experimental groups at baseline (Table 3). The estimated daily intake of non-digestible dietary fibres, starch, protein, cholesterol and carbohydrates were not changed from baseline to end point. The estimated daily total fat intake was significantly increased in the Control group (15 of the 20 participants showed increased fat intake), but this change was not significantly different when compared to the Cod group and the Salmon group. The combined intake of the macronutrients fat, protein and carbohydrates was not changed within any of the group or between the groups (*p* ANCOVA for group comparisons was 0.29, data not presented).

**Table 3:** Estimated daily dietary intake of dietary fibre, starch, fat, protein, cholesterol and carbohydrates based on 5 days dietary records at baseline and after 8 weeks*

	Baseline		8 weeks		*P*^†^	*P*^‡^
	Median	25th, 75th percentile	Median	25th, 75th percentile		
Non-digestible dietary fibre (g/day)						
Cod group	20.0	16.2,24.5	20.6	17.4,27.3	0.43	0.67
Salmon group	18.7	15.5,21.8	18.8	16.3,23.5	0.78	
Control group	17.9	13.8,20.9	17.7	13.8,25.2	0.31	
Starch (g/day)						
Cod group	115	82,141	107	88,136	0.78	0.81
Salmon group	114	93,143	96	80,133	0.41	
Control group	133	99,168	102	95,143	0.21	
Fat (g/day)						
Cod group	90	68,115	83	66,108	0.45	0.096
Salmon group	94	64,120	93	84,120	0.14	
Control group	91	74,113	91	81,126	0.036	
Protein (g/day)						
Cod group	94	84,122	100	74,114	0.86	0.88
Salmon group	86	77,104	92	83,98	0.85	
Control group	92	80,106	91	72,122	0.83	
Cholesterol (mg/day)						
Cod group	411	260,491	400	305,516	0.88	0.79
Salmon group	372	287,602	367	324,474	0.47	
Control group	308	207,438	363	223,502	0.30	
Carbohydrates (g/day)						
Cod group	203	168,248	181	162,225	0.18	0.49
Salmon group	197	167,231	188	144,233	0.74	
Control group	209	164,272	195	141,281	0.90	

Medians and 25th and 75th percentiles

^*^No differences were seen between the groups at the baseline (Kruskal–Wallis test). Results are presented for 22 participants in the Cod group, 23 participants in the Salmon group and 20 participants in the Control group

^†^Within-group changes are tested using the Wilcoxon’s signed-ranks test

^‡^Changes within Cod group, Salmon group and Control group are compared using analysis of covariance (ANCOVA) with adjustment for baseline values after log transformation

In the subgroups of participants that collected all faeces produced for 72 h before the baseline and end point visits, the median daily total faeces wet weight, dry weight, cholesterol, total fat (measured as fatty acids) or total bile acids outputs were not different between the groups at baseline and did not change from baseline to end point within any of the groups (Table 4).

**Table 4:** Faecal output at baseline and after 8 weeks*

	Baseline		8 weeks		*P*†	*P*‡
	Median	25th, 75th percentile	Median	25th, 75th percentile		
Total wet weight of faeces, g/day						
Cod group	189	107,238	183	129,227	0.65	0.53
Salmon group	133	80,228	131	68,190	0.61	
Control group	145	81,198	130	77,197	0.53	
Total dry weight of faces, g/day						
Cod group	42	27,63	39	29,58	0.65	0.61
Salmon group	34	16,51	27	23,30	0.12	
Control group	29	22,39	20	16,37	0.73	
Cholesterol, µmol/day						
Cod group	829	532,1139	938	703,1255	0.65	0.56
Salmon group	699	276,1592	524	258,941	0.14	
Control group	760	347,2201	538	253,1460	0.24	
Total fatty acids, g/day						
Cod group	6.47	3.94,12.52	7.21	4.18,9.33	0.80	0.61
Salmon group	5.63	3.40,8.22	5.88	3.49,9.48	0.31	
Control group	4.24	3.16,7.79	4.62	2.23,8.33	0.53	
Total bile acids, µmol/day						
Cod group	199	183,208	205	188,209	0.14	0.63
Salmon group	198	182,204	203	192,210	0.67	
Control group	193	171,210	197	163,211	0.72	

Medians and 25th and 75th percentiles

^*^No differences were seen between the groups at the baseline (Kruskal–Wallis test). Results are presented for 10 participants in the Cod group, 15 participants in the Salmon group and 12 participants in the control group

^†^Within-group changes are tested using the Wilcoxon’s signed-ranks test

^‡^Changes within the Cod group, Salmon group and Control group are compared using the analysis of covariance (ANCOVA) with adjustment for baseline values after log transformation

### Gut microbiota

**Fig. 2:**
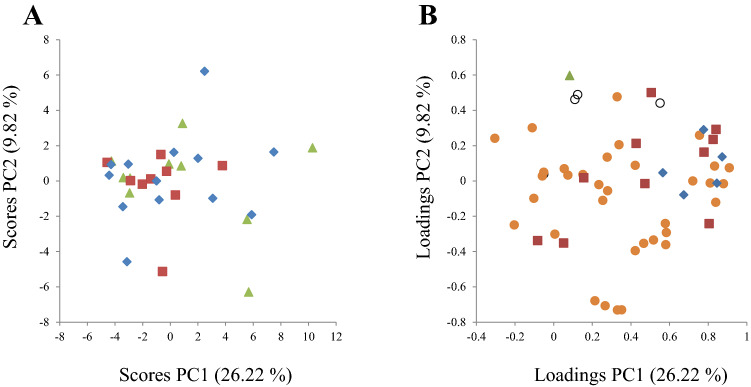
Scores (**a**) and loadings (**b**) from the first two principal components (PC1 and PC2) obtained from principal component analysis using centred and standardised gut bacteria counts in faeces at baseline for *N* = 9 subjects in the Cod group, *N* = 13 subjects in the Salmon group, and *N* = 11 subjects in the Control group. The score plot (**a**) shows the experimental groups (blue diamond; Cod group, red squares; Salmon group, green triangles; Control group). The loading plot (**b**) show bacterial signals by phylum (open black circles; *Actinobacteria*, red squares, *Bacteroidetes*, orange circles; *Firmicutes*, blue diamond; *Proteobacteria*, green triangle; *Verrucomicrobia*)

The signals for 54 gut microbiota DNA probes in total 72 h faeces collected at baseline and at end point were compared for 33 participants (*N* = 9 in Cod group, *N* = 13 in Salmon group and *N* = 11 in Control group) using PCA. For the baseline analyses, the PCA score plot revealed no separation between the dietary groups by the two first principal components (PC1 explained 26.22% and PC2 explained 9.82% of the variation in the dataset, Fig. 2a). The loading plot showed no clear grouping of the bacteria at baseline (Fig. 2b). The PCA showed no relation of the microbiota profile with participants’ age, sex, BMI, percent body fat, percent muscle mass, dietary intake (fibre, starch, fat, protein, carbohydrates, cholesterol), serum lipids or NEFA, serum CRP, whole blood HbA1c, stool content of dry mass and moisture, and daily faecal output of fat, cholesterol and total bile acids at baseline (data not presented).

**Fig. 3:**
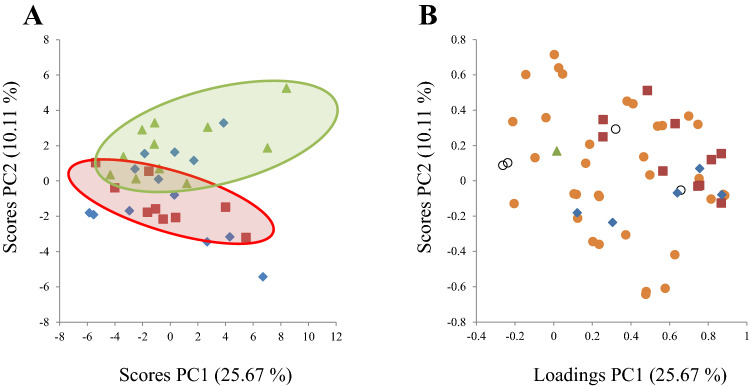
Scores (**a**) and loadings (**b**) from the first two principal components (PC1 and PC2) obtained from principal component analysis using centred and standardised gut bacteria counts in faeces at end point for *N* = 9 subjects in the Cod group, *N* = 13 subjects in the Salmon group, and *N* = 11 subjects in the Control group. The score plot (**a**) shows the experimental groups (blue diamond; Cod group, red squares; Salmon group encircled by a red ellipse, green triangles; Control group encircled by a green ellipse). The loading plot (**b**) show bacterial signals by phylum (open black circles; *Actinobacteria*, red squares, *Bacteroidetes*, orange circles; *Firmicutes*, blue diamond; *Proteobacteria*, green triangle; *Verrucomicrobia*)

In the PCA for end point data, the participants showed a propensity to cluster according to their experimental group affiliation (PC1 and PC2 explained 25.67 and 10.11%, respectively, of the variation in the dataset, Fig. 3a). The Salmon group was almost completely separated from the Control group, whereas the Cod group showed greater similarity to the Salmon group than to the Control group. The loading plot revealed that the separation was in part due to a trend of lower counts for bacteria that belong to the *Bacteroidales* order in the *Bacteroidetes* phylum and the *Clostridiales* order of the *Firmicutes* phyla, and higher counts for a few bacteria in the *Firmicutes* phylum including the *Selenomonadales* order in the Cod and Salmon groups when compared to the Control group (Fig. 3b).

### Serum lipids

Serum concentrations of total cholesterol, LDL-cholesterol, triacylglycerol, NEFA and total bile acids were not changed from baseline to end point within any of the experimental groups, and no differences were seen between the groups for changes over time (Table 5). Serum HDL-cholesterol was reduced after 8 weeks in the Control group, but this change was not significantly different when compared to the Cod group and the Salmon group.

**Table 5:** Serum fasting concentrations of lipids, NEFA and total bile acids at baseline and after 8 weeks*

	Baseline		8 weeks		*P*†	*P*‡
	Median	25th, 75th percentile	Median	25th, 75th percentile		
Serum total cholesterol, mmol/l						
Cod group	5.7	4.5,6.1	5.5	4.3,6.4	0.84	0.26
Salmon group	5.0	4.4,5.5	4.8	4.2,5.4	0.10	
Control group	5.2	4.4,5.5	5.0	4.3,5.4	0.19	
Serum HDL-cholesterol, mmol/l						
Cod group	1.3	1.0,1.5	1.3	0.9,1.5	0.59	0.21
Salmon group	1.3	1.2,1.4	1.3	1.0,1.4	0.48	
Control group	1.3	1.1,1.6	1.2	1.0,1.5	0.0075	
Serum LDL-cholesterol, mmol/l						
Cod group	3.5	2,6,4.5	3.6	2.6,4.4	0.30	0.39
Salmon group	3.2	2.6,3.6	3.1	2.4,3.5	0.30	
Control group	3.4	2.5,3.6	3.2	2.5,3.5	0.97	
Serum triacylglycerols, mmol/l						
Cod group	1.26	1.08,1.93	1.20	0.91,1.64	0.78	0.33
Salmon group	0.98	0.76,1.45	1.00	0.73,1.53	0.76	
Control group	1.35	0.90,1.69	1.41	0.91,1.83	0.32	
Serum NEFA, mmol/l						
Cod group	0.50	0.39,0.70	0.52	0.37,0.64	0.88	0.92
Salmon group	0.51	0.43,0.71	0.48	0.37,0.56	0.38	
Control group	0.56	0.35,0.66	0.52	0.45,0.63	0.89	
Serum total bile acids, µmol/l						
Cod group	1.0	0.7,2.3	1.5	0.5,2.0	0.81	0.41
Salmon group	1.0	0.6,3.0	1.0	0.8,2.0	0.70	
Control group	1.0	0.8,3.0	2.5	1.0,3.0	0.42	

Medians and 25th and 75th percentiles

^*^No differences were seen between the groups at the baseline (Kruskal –Wallis test). Results are presented for 22 participants in the Cod group, 22 participants in the Salmon group and 19 participants in the control group. NEFA, non-esterified fatty acids

^†^Within-group changes are tested using the Wilcoxon’s signed-ranks test

^‡^Changes within Cod group, Salmon group and Control group are compared using the analysis of covariance (ANCOVA) with adjustment for baseline values after log transformation

### Correlations between estimated dietary intakes, faecal outputs and serum lipid concentrations

The combined baseline and end point data sets were analysed using Spearman’s two-tailed correlation analysis (Table 6). The dietary intakes of fibre, starch, fat, proteins, cholesterol and carbohydrates were correlated with wet and dry faecal weight, with the strongest correlation between fibre intake and faecal dry weight (Spearman’s correlation 0.418, *p* = 2.4 × 10^–4^). Faecal cholesterol output correlated with intakes of starch and carbohydrates, and faecal fat output correlated with intakes of fibre, fat, proteins and cholesterol, whereas total bile acid faecal output was not significantly correlated to dietary intakes. Serum cholesterol, HDL-cholesterol, LDL-cholesterol, triacylglycerols and total bile acids concentrations were not correlated to faecal outputs, but serum NEFA concentration was inversely correlated to faecal wet and dry weights and faecal outputs of fat and total bile acids. The strongest associations for faecal outputs of total cholesterol and fat were seen for faecal wet and dry weights, with *p* < 10^–8^, however correlations were not statistically significant between faecal weights and faecal total bile acids output. The correlation between wet and dry daily faecal weights were 0.850 (*p* = 1.1 × 10^–21^).

**Table 6:** Spearman’s correlations of estimated daily intakes, faecal daily outputs and serum concentrations of lipids and total bile acids, combined for baseline and end point data sets

	Estimated daily intake						Faecal daily output					Serum concentrations				
	Fibre	Starch	Fat	Protein	TC	CH	Wet weight	Dry weight	TC	Fat	TBA	TC	HDL	LDL	TAG	NEFA
Estimated daily intake (*N* = 130)																
Starch	0.375^***^															
Total fat	0.201^*^	0.302^***^														
Protein	0.269^**^	0.356^***^	0.699^***^													
TC	− 0.010	− 0.061	0.479^***^	0.612^***^												
CH	0.439^***^	0.753^***^	0.304^***^	0.233^**^	− 0.140											
Faecal daily output (*N* = 74)																
Wet weight	0.335^**^	0.322^**^	0.307^**^	0.393^***^	0.267^*^	0.336^**^										
Dry weight	0.418^***^	0.368^**^	0.326^**^	0.368^**^	0.214	0.353^**^	0.850^***^									
TC	0.042	0.317^**^	0.014	0.078	− 0.086	0.368^**^	0.739^***^	0.604^***^								
Fat	0.431^***^	0.136	0.294^*^	0.358^**^	0.325^**^	0.150	0.713^***^	0.778^***^	0.368^**^							
TBA	− 0.034	0.176	0.070	0.087	− 0.034	0.204	− 0.175	0.001	− 0.187	− 0.224						
Serum fasting concentrations (*N* = 130)																
TC	− 0.152	− 0.231^**^	− − 0.009	0.088	0.310^***^	− 0.210^*^	0.024	0.053	− 0.056	− 0.003	0.119					
HDL	− 0.054	− 0.130	− 0.070	− 0.166	0.011	− 0.155	0.006	− 0.057	− 0.084	0.073	− 0.203	0.005				
LDL	− 0.115	− 0.209^*^	− 0.006	0.085	0.281^**^	− 0.182^*^	− 0.004	0.044	− 0.099	− 0.011	0.148	0.955^***^	− 0.150			
TAG	− 0.104	0.072	0.021	0.109	0.143	0.085	0.069	0.073	0.137	− 0.056	0.159	0.453^***^	− 0.381^***^	0.373^***^		
NEFA	0.017	− 0.073	− 0.238^**^	− 0.181^*^	− 0.202^*^	− 0.103	− 0.233^*^	− 0.252^*^	− 0.105	− 0.262^*^	0.245^*^	0.043	0.018	0.006	0.092	
TBA	− 0.015	0.070	− 0.004	− 0.097	− 0.109	− 0.030	− 0.071	0.058	0.065	− 0.009	0.101	− 0.121	− 0.073	− 0.156	0.042	− 0.015

*Fibre* non-digestible dietary fibre, *TC* total cholesterol, *CH* carbohydrates, *TBA* total bile acids, *HDL* HDL-cholesterol, *LDL* LDL-cholesterol, *TAG* triacylglycerols, *NEFA* Non-esterified fatty acids

^*^Correlation is significant at the 0.05 level (2-tailed)

^**^Correlation is significant at the 0.01 level (2-tailed)

### Correlations between biomarkers of Cod and Salmon intake and gut microbiota

We recently found that serum and urine concentrations of TMAO and 1-MeHis were promising biomarkers of fish intake, as both serum and urine concentrations of TMAO and 1-MeHis were significantly increased after cod and salmon intake, respectively, in samples from participants in the present study [28]. Here, we explored the correlations between these biomarkers and gut microbiota signals, and bacteria showing at least one statistically significant correlation are presented in Table 7. Serum and urine TMAO concentrations were positively correlated with the *Akkermansia muciniphila* signal at baseline, whereas these correlations were not siginficant at end point. Serum TMAO concentration was inversely correlated with *Clostridium methylpentosum* at baseline, but not at end point. Urine TMAO concentration was positively correlated with *Bacteroides* sp. at baseline and negatively correlated with *Bacteroides* sp. at end point, but only baseline urine TMAO concentration correlated with *Proteobacteria*.

**Table 7:** Spearman’s correlations of serum and urine concentrations of trimethylamine N-oxide (TMAO) and 1-methylhistidine (1-MeHis) with gut microbiota combined for Cod group, Salmon group and Control group, showing only correlations where at least one bacterial probe is significantly correlated with TMAO or 1-MeHis

Counts	Serum (µmol/l)		Urine (µmol/mmol creatinine)	
	TMAO	1-MeHis	TMAO	1-MeHis
Baseline				
*Akkermansia muciniphila*	0.416^*^	0.366^*^	0.375^*^	0.084
*Bacteroides* sp.	0.200	− 0.068	0.372^*^	− 0.066
*Clostridium methylpentosum*	− 0.350^*^	0.096	− 0.073	0.218
*Coprobacillus_cateniformis*	0.229	− 0.085	0.047	− 0.011
*Eubacterium rectale*	− 0.115	0.086	− 0.071	0.141
*Lachnosp* Incertae Sedis	− 0.186	0.382^*^	− 0.127	0.331
*Mycoplasma hominis*	0.253	0.100	0.298	0.232
*Phascolarctobacterium faecium*	0.129	0.437^*^	0.212	0.222
*Proteobacteria*	0.201	0.166	0.358^*^	0.064
*Staphylococcus epidermidis*	− 0.213	0.125	− 0.294	0.136
*Streptococcus agalactiae*	− 0.297	− 0.117	− 0.148	0.085
Endpoint				
*Akkermansia muciniphila*	0.306	− 0.022	− 0.142	− 0.106
*Bacteroides* sp.	− 0.128	0.103	− 0.417^*^	− 0.087
*Clostridium methylpentosum*	0.135	− 0.077	0.106	− 0.097
*Coprobacillus cateniformis*	− 0.025	− 0.079	− 0.163	− 0.415^*^
*Eubacterium rectale*	− 0.130	− 0.384^*^	− 0.286	− 0.482^**^
*Lachnosp* Incertae Sedis	− 0.059	− 0.214	− 0.265	− 0.338
*Mycoplasma hominis*	0.080	0.151	− 0.040	0.371^*^
*Phascolarctobacterium faecium*	0.201	0.272	− 0.223	− 0.216
*Proteobacteria*	0.089	− 0.062	0.126	0.034
*Staphylococcus epidermidis*	− 0.121	− 0.321	− 0.307	− 0.364^*^
*Streptococcus agalactiae*	− 0.233	− 0.310	− 0.282	− 0.513^**^

^*^Correlation is significant at the 0.05 level (two-tailed)

^**^Correlation is significant at the 0.01 level (two-tailed)

Baseline serum concentration of 1-MeHis was positively correlated with *Akkermansia muciniphila* and *Lachnosp* Incertae Sedis, *Phascolarctobacterium faecium*, whereas no correlations with gut microbiota were seen for urine 1-MeHis at baseline. At end point, serum 1-MeHis was negatively correlated with *Eubacterium rectale* and urine 1-MeHis was negatively correlated with *Coprobacillus cateniformis*, *Eubacterium rectale*, *Staphylococcus epidermidis* and *Streptococcus agalactiae*, and positively correlated with *Mycoplasma hominis*.

## Discussion

In the present study, we investigated the effects of high intake of cod or salmon on the gut microbiota profile. We observed that a weekly intake of 750 g of cod or salmon changed the microbiota composition, but did not change faecal output and serum concentrations of lipids and total bile acids.

Dietary proteins are substrates for microbial energy metabolism in the colon [2, 3] and may affect gut microbiota profile. Here, we show that high intake of cod or salmon resulted in a separation of bacteria by the PCA, with lower counts for bacteria in the *Bacteroidetes* phylum and the *Clostridiales* order of the *Firmicutes* phyla, and higher counts for bacteria in the *Selenomonadales* order in the *Firmicutes* phylum when compared to the Control group. The separation from Control group was almost complete for participants in the Salmon group, and less clear for those in the Cod group. Although the first two components from the PCA explained merely 35.78% of the variation in the dataset, this observed separation between the experimental groups are of potentially great interest. A higher abundance of *Bacteroidetes* has been found in patients with type 2 diabetes [40], and the present finding of lower counts of *Bacteroidetes* especially in the Salmon group fits nicely with our previous report of improved postprandial glucose regulation after high salmon intake [31]. Lower numbers of *Bacteroides* spp. have also been observed in fish fermentations compared to chicken in vitro for human faeces [41]. Similar findings have been reported for gut bacteria in the caecum in Sprague–Dawley rats fed fish as the sole protein source, with lower abundance of *Bacteroidetes* however with higher abundance of *Firmicutes* [27], whereas intake of lean seafood or n-3 PUFA supplementation did not affect gut microbiome composition [25, 26]. The differences in gut microbiota profile between the experimental groups in the present study could be a consequence of the high intake of cod or salmon in the fish groups, but it may also be due to the higher intake of meat in the Control group where nearly 100% of dinners contained red or white meat as opposed to around only 29% of the dinners in the fish-eating groups [28]. Since there is no consensus regarding what can be considered a healthy human gut microbiome [1], an interpretation of the present findings cannot be made beyond stating that high intake of cod or salmon has the capacity to alter the gut microbiome profile compared to a Control group consuming red or white meat.

The faecal microbiota richness and variance of bacteria is strongly influenced by stool moisture, which in turn is determined by transit time and residence time in the colon and rectum [1]. Fibre intake, especially non-digestible fibre, and physical activity are associated with shorter intestinal transit time and increased gastrointestinal motility, leading to a higher water content in the stool [42, 43]. Also total food intake, body weight and diets are important determinants for total stool mass [42]. The medians for measured daily faecal wet weight and outputs of dry mass, fat, cholesterol and total bile acids were within the normal ranges and indicated healthy gastrointestinal function in our participants [42]. Non-digestible fibres undergo minimal changes in the digestive tract and have a high water holding capacity and thus increase faecal mass, but also digestible fibres can cause an increase in faecal weights since the presence of fermentable substrates will stimulate proliferation of bacteria and results in soft, bulky and water-retaining stools [42]. In line with this, the unchanged faecal wet and dry weights (from two 72 h faecal collection periods) during the course of the present trial corresponds well with the unaltered intake of dietary fibre and the unchanged levels of physical activity [31]. The significant correlation between estimated dietary fibre intake and measured faecal dry mass output (Spearman’s correlation 0.418, *p* = 2.4 × 10^–4^) suggests that both the registration of food intake and the collection and analysis of faeces were successful. Since no changes were seen in wet and dry faecal mass within any of the experimental groups, we conclude that the differences in gut microbiota profile between the groups were due to genuine differences in composition and were not a result of a ‘dilution effect’.

The role of faecal output in overweight and obesity is unclear, but the removal of excess energy by increased faecal excretion of lipids represents an attractive target and is the principle of weight loss drugs such as Orlistat. The faecal fat consists of undigested fat from dietary intake, bacteria and shredded epithelial cells [42], and the faecal fat excretion in healthy persons is ≤ 7 g per day when the daily fat intake from the diet is between 50 and 150 g [44]. In the present study, the estimated median fat intake from food diaries at baseline and end point was 90.5 (interquartile range 72–114) g/day and the measured median faecal fat excretion for these time points was 5.5 (interquartile range 3.5–8.9) g/day, and we found a weak but significant correlation between fat intake and faecal fat excretion (Spearman’s correlation 0.294, *p* = 0.012). Diets that are rich in dietary fibres will increase the faecal output of fat by reducing the absorption capacity in the gut [42], and in line with this we found a strong correlation between dietary fibre intake and faecal fat output (Spearman’s correlation 0.431, *p* = 1.4 × 10^–4^). Fat contributed to a median 19% (interquartile range 14–23) of the faecal dry weight in the present study and these parameters were strongly correlated (Spearman’s correlation 0.778, *p* = 3.6 × 10^–16^). Also exercise may affect gastrointestinal and gallbladder motility [43]; therefore, since the participants in this study were instructed to not change their eating habits except for the mandatory fish intake in the Cod group and the Salmon group and no-fish intake in the Control group, together with no change in the level of physical activity during the intervention period [31], it is not surprising that we did not observe any changes in faecal fat excretion.

The circulating concentration of cholesterol is controlled by the rate of cholesterol biosynthesis regulated by HMG-CoA reductase, by the uptake of LDL-particles facilitated by LDL-receptors and through faecal excretion of cholesterol and bile acids. Bile formation is essential for total body cholesterol balance, as biliary excretion of cholesterol and conversion of cholesterol to bile acids are the principal routes of cholesterol excretion and catabolism, and the gut microbiota plays a key role in the enterohepatic circulation of bile acids [6, 7]. An increase in faecal bile acid excretion would enhance the biosynthesis of cholesterol and trigger an increased production of bile acids and up-regulate LDL-receptors leading to lower circulating LDL-cholesterol concentrations. We recently investigated the effects of replacing 25 wt% of casein in the regular AIN-93G diet [45] with corresponding amounts of proteins from cod or salmon fillets on cholesterol metabolism in obese Zucker fa/fa rats. Cod fillet feeding resulted in lower serum total, HDL- and LDL-cholesterol concentrations and lower hepatic mRNA expressions of HMG-CoA reductase and LDL-receptor, without affecting serum and faecal total bile acid concentration, faecal and liver cholesterol amounts in faeces, or liver cholesterol 7 alpha-hydroxylase mRNA concentration in these rats [16]. These findings indicate that the lower circulating cholesterol concentration was not regulated through increased faecal output of cholesterol and/or bile acids, but was a result of lower endogenous production of cholesterol in rats fed cod fillet. Similar results were seen when obese Zucker fa/fa rats were fed salmon fillet as 25 wt% of the diet; serum concentrations of total, HDL- and LDL-cholesterol were lower in these rats compared to those fed the regular AIN-93G diet, with no effect on faecal excretion of cholesterol and total bile acids [17]. In contrast to our findings in rats, we observed no effects on serum cholesterol after high intake of cod or salmon in the present clinical trial. However, the observation that faecal outputs of cholesterol and total bile acids were not affected by high cod or salmon intake in our participants with obesity/overweight corresponded well with previous studies in rats fed cod or salmon fillet [16, 17].

TMAO concentrations in serum and urine are affected by gut microbiota production of trimethylamine from dietary precursors, dietary intake of TMAO and/or its precursors, and kidney function [46, 47]. Participants in the present study appeared to have normal kidney function since serum creatinine and urine albumin concentrations were within normal range both at start and the end of the intervention period, and TMAO seems to be a good biomarker for cod intake in this study population with a significant increase in TMAO concentrations in serum and urine in this dietary group after 8 weeks intervention [28]. Trimethylamine, the bacterial precursor for TMAO, is generated by gut microbiota through three pathways, either from choline, trimethylglycine (betaine), ergothioneine, dimethylglycine or carnitine, which are found in common foodstuffs such as seafood, terrestrial meat, egg, dairy products, vegetables, mushrooms, kidneys and liver; from dietary L-carnitine that may be converted to gamma-butyrobetaine [46]; or by reduction of TMAO by the gut microbiota (predominantly *Enterobacteriaceae* in the phyla *Proteobacteria*) [48]. Trimethylamine is transported to the liver for oxidation to TMAO by flavin-containing monooxygenases [46]. Various bacterial phyla including *Actinobacteria*, *Bacteroidetes*, *Firmicutes* and *Proteobacteria* are associated with the first pathway (mentioned above) in the human colon [46], whereas especially *Akkermansia muciniphila* has been demonstrated to be associated with the carnitine–gamma-butyrobetaine–TMAO pathway [49]. In the present study, we found positive correlations between serum and urine TMAO concentrations and the *Akkermansia muciniphila* signal (phylum: *Verrucomicrobia*) at baseline, and correlations were also found between serum TMAO concentration and *Clostridium methylpentosum* (phylum: *Firmicutes*), and between urine TMAO concentration and *Bacteroides* sp. (phylum: *Bacteroidetes*) and unspecified bacteria of the phylum *Proteobacteria*. Thus, TMAO may have been produced by either of the microbiota pathways for trimethylamine at baseline. The hepatic production of TMAO from gut microbiota-originating trimethylamine is small compared to the TMAO contribution from fish intake [46]. Therefore, it is not surprising that the correlations between TMAO and gut bacteria for our study population were fewer (the only correlation was for urine TMAO concentration with *Bacteroides* sp*.*) after interventions with cod or salmon in two of the three experimental groups, since TMAO predominately originated from fish intake and not microbial production.

1-MeHis, a potential biomarker for salmon intake [28], has not been described as a metabolite of or substrate for the gut microbiota. We have recently shown that serum and urine concentrations of 1-MeHis were significantly increased in the Salmon group in the present study [28]. 1-MeHis from diet is not reutilised for protein synthesis or metabolised, and it is estimated that 90% of 1-MeHis from the diet is excreted in urine as 1-MeHis in humans [50]. At end point, urine concentration of 1-MeHis was negatively correlated with a few bacterial species, mainly in the *Firmicutes* phylum, with the strongest negative correlations with *Eubacterium rectale* (class: *Clostridia*) and *Streptococcus agalactiae* (class: *Bacilli*), and with positive correlation with *Mycoplasma hominis*. Since little information is available regarding the consumption and production of 1-MeHis by gut microbiota, it is difficult to conclude if these correlations are a result of changes in consumption or production of 1-MeHis by gut microbiota or simply non-causal associations. The described correlations between urine 1-MeHis concentration and gut microbiota correspond well with the results from PCA using end point data, thus urine 1-MeHis concentration may be a useful biomarker for salmon intake in future studies when interpreting the effects of diets on gut microbiota in humans, as an alternative or an addition to food diaries or food-frequency questionnaires.

We have recently shown that a high intake of salmon, but not of cod, for 4 weeks reduced triacylglycerol and increased HDL-cholesterol serum concentrations in healthy, normal weight adults [13]. This is in sharp contrast to findings in adults with overweight or obesity in the present study, where we found no effect of high cod or salmon intake on any of the measured serum lipid concentrations. This may be a consequence of larger heterogeneity in regard to factors such as age and percentage of body fat and muscle mass in the latter study.

The present study has some strengths and limitations. A strength is the use of 72 h faeces collection at baseline and end point, rather than using spot samples since the day to day variation in the faecal mass can vary widely for the same individual [1]. Also, faecal mass is affected by diet and physical activity [42], and gut microbiota is affected by medications including antibiotics [51] and metformin [52]. Our study participants did not change their diet except the mandatory intake of cod or salmon in the fish-eating groups or their physical activity, and none of the participants reported using antibiotics, probiotics, prebiotics or antidiabetics at baseline or end point, or at any time during the intervention period. Limitations for this study include shortcomings associated with the participants’ faeces collection, such as failing to collect all produced stool over the 72 h periods and immediately depositing the filled containers in the freezer as instructed. The choice of analysing only 54 bacterial DNA probes using a method originally developed for identifying dysbiosis [33] can be criticised; still, the findings are of interest since the Salmon group and the Control group were partially separated by PCA. Complete gut bacteria characterisation should be conducted in future studies with similar design. The intakes of prebiotics such as polyphenols and inulin found in fruits, vegetables and berries may also affect gut microbiota [1, 53], but were not estimated in the present study. The sample size was relatively small, and a type II error may mask significant effects of the intervention in both gut microbiota and faecal output and serum concentrations of lipids and total bile acids due to low statistical power.

To conclude, despite the small sample size and the limited number of bacterial probes used, this study is unique since it demonstrates that a high intake of cod or salmon fillet may modulate gut microbiota in adults with overweight/obesity without known gastrointestinal disease. The high intake of cod or salmon did not affect faecal output or serum lipids and bile acids.

## Electronic supplementary material

Below is the link to the electronic supplementary material.

Supplementary file1 (DOCX 22 kb)
