# Supplementary material for: Effects of high intake of cod or salmon on gut microbiota profile, faecal output and serum concentrations of lipids and bile acids in overweight adults: a randomised clinical trial
Source: Eur J Nutr. 2020 Oct 27;60(4):2231–48. doi: 10.1007/s00394-020-02417-8 (PMC8137623; doi:10.1007/s00394-020-02417-8)
Supplement: Supplementary file 1 — Supplementary file1 (DOCX 22 kb) [file 394_2020_2417_MOESM1_ESM.docx]

**Supplemental Table: Overview of the 54 bacterial DNA probes used**

| **Phylum** | **Class** | **Order** | **Family** | **Genus** | **Specific species/comments** |
| --- | --- | --- | --- | --- | --- |
| 28 species specific probes: | |  |  |  |  |
| *Actinobacteria* | *Coriobacteriia* | *Coriobacteriales* | *Corinobacteriaceae* | *Atopobium* | *Atopobium rimae* |
| *Bacteroidetes* | *Bacteroidia* | *Bacteroidales* | *Bacteroidaceae* | *Bacteroides* | *Bacteroides fragilis* |
| *Bacteroidetes* | *Bacteroidia* | *Bacteroidales* | *Bacteroidaceae* | *Bacteroides* | *Bacteroides stercoris* |
| *Bacteroidetes* | *Bacteroidia* | *Bacteroidales* | *Bacteroidaceae* | *Bacteroides* | *Bacteroides zoogleoformans* |
| *Bacteroidetes* | *Bacteroidia* | *Bacteroidales* | *Bacteroidaceae* | *Prevotella* | *Prevotella nigrescens* |
| *Bacteroidetes* | *Bacteroidia* | *Bacteroidales* | *Porphyromonadaeae* | *Parabacteroides* | *Parabacteroides johnsonii* |
| *Bacteroidetes* | *Bacteroidia* | *Bacteroidales* | *Rikenellaceae* | *Alistipes* | *Alistipes onderdonkii* |
| *Firmicutes* | *Bacilli* | *Bacillales* | *Bacillaceae* | *Bacillus* | *Bacillus megaterium* |
| *Firmicutes* | *Bacilli* | *Bacillales* | *Staphylococcaceae* | *Staphylococcus* | *Staphylococcus epidermidis* |
| *Firmicutes* | *Bacilli* | *Lactobacillales* | *Lactobacillaceae* | *Lactobacillus* | *Lactobacillus acidophilus* |
| *Firmicutes* | *Bacilli* | *Lactobacillales* | *Lactobacillaceae* | *Lactobacillus* | *Lactobacillus ruminis* |
| *Firmicutes* | *Bacilli* | *Lactobacillales* | *Streptococcaceae* | *Streptococcus* | *Streptococcus agalactiae* |
| *Firmicutes* | *Clostridia* | *Clostridiales* | *Clostridiaceae* | *Anaerotruncus* | *Anaerotruncus colihominis* |
| *Firmicutes* | *Clostridia* | *Clostridiales* | *Clostridiaceae* | *Clostridium* | *Clostridium methylpentosum* |
| *Firmicutes* | *Clostridia* | *Clostridiales* | *Eubacteriaceae* | *Eubacterium* | *Eubacterium hallii* |
| *Firmicutes* | *Clostridia* | *Clostridiales* | *Eubacteriaceae* | *Eubacterium* | *Eubacterium rectale* |
| *Firmicutes* | *Clostridia* | *Clostridiales* | *Eubacteriaceae* | *Eubacterium* | *Eubacterium siraeum* |
| *Firmicutes* | *Clostridia* | *Clostridiales* | *Peptococcaceae* | *Desulfitispora* | *Desulfitispora alkaliphila* |
| *Firmicutes* | *Clostridia* | *Clostridiales* | *Ruminococcaceae* | *Ruminococcus* | *Ruminococcus bromii* |
| *Firmicutes* | *Clostridia* | *Clostridiales* | *Ruminococcaceae* | *Ruminococcus* | *Ruminococcus gnavus* |
| *Firmicutes* | *Clostridia* | *Clostridiales* | *unclassified Clostridiales* |  | *[Bacteroides] pectinophilus* |
| *Firmicutes* | *Erysipelotrichi* | *Erysipelotrichales* | *Erysipelotrichidae* | *Catenibacterium* | *Catenibacterium mitsuokai* |
| *Firmicutes* | *Erysipelotrichi* | *Erysipelotrichales* | *Erysipelotrichidae* | *Coprobacillus* | *Coprobacillus cateniformis* |
| *Firmicutes* | *Mollicutes* | *Mycoplasmatales* | *Mycoplasmataceae* | *Mycoplasma* | *Mycoplasma hominis* |
| *Firmicutes* | *Negativicutes* | *Acidaminococcales* | *Acidaminococcaceae* | *Phascolarctobacterium* | *Phascolarctobacterium faecium* |
| *Firmicutes* | *Negativicutes* | *Selenomonadales* | *Veillonellaceae* | *Dialister* | *Dialister invisus* |
| *Proteobacteria* | *Gammaproteobacteria* | *Pseudomonadales* | *Moraxellaceae* | *Acinetobacter* | *Acinetobacter junii* |
| *Verrucomicrobia* | *Verrucomicrobiaea* | *Verrucomicrobiales* | *Akkermansiaceae* | *Akkermansia* | *Akkermansia muciniphila* |
|  |  |  |  |  |  |
| 18 probes detected bacteria at genus level: | | |  |  |  |
| *Actinobacteria* | *Actinobacteria* | *Actinomycetales* | *Actinomycetaceae* | *Actinomyces* |  |
| *Actinobacteria* | *Actinobacteria* | *Bifidobacteriales* | *Bifidobacteriaceae* | *Bifidobacterium* |  |
| *Bacteroidetes* | *Bacteroidia* | *Bacteroidales* | *Bacteroidaceae* | *Bacteroides* |  |
| *Bacteroidetes* | *Bacteroidia* | *Bacteroidales* | *Bacteroidaceae* | *Bacteroides* | *Bacteroides* sp 2_2_4/*ovatus*/sp D1 |
| *Bacteroidetes* | *Bacteroidia* | *Bacteroidales* | *Porphyromonadaeae* | *Parabacteroides* |  |
| *Bacteroidetes* | *Bacteroidia* | *Bacteroidales* | *Rikenellaceae* | *Alistipes* |  |
| *Firmicutes* | *Bacilli* | *Lactobacillales* | *Lactobacillales* | *Lactobacillus* |  |
| *Firmicutes* | *Bacilli* | *Lactobacillales* | *Streptococcaceae* | *Streptococcus* | *Streptococcus* (alpha-haemolytic) |
| *Firmicutes* | *Bacilli* | *Lactobacillales* | *Streptococcaceae* | *Streptococcus* | *Streptococcus* (beta-haemolytic group A and B, alpha-haemolytic) |
| *Firmicutes* | *Bacilli* | *Lactobacillales* | *Streptococcaceae* | *Streptococcus* | *Streptococcus sanguinis* and *thermophilus* |
| *Firmicutes* | *Bacilli* | *Lactobacillales* | *Streptococcaceae* | *Streptococcus* | *Streptococcus thermophilus* pluss others |
| *Firmicutes* | *Clostridia* | *Clostridiales* | *Clostridiaceae* | *Clostridium* | *Clostridium* sp L2-50 |
| *Firmicutes* | *Erysipelotrichia* | *Erysipelotrichales* | *Erysipelotrichaceae* | *Eubacterium* | *Eubacterium biforme* (signal on several species) |
| *Firmicutes* | *Clostridia* | *Clostridiales* | *Lachnospiraceae* | *Dorea* | *Dorea formicigenerans* (*Dorea longicatena*) |
| *Firmicutes* | *Clostridia* | *Clostridiales* | *Ruminococcaceae* | *Faecalibacterium* | *Faecalibacterium cf prausnitzi* |
| *Firmicutes* | *Negativicutes* | *Selenomonadales* | *Veillonellaceae* | *Dialister* |  |
| *Proteobacteria* | *Gammaproteobacteria* | *Enterobacterales* | *Enterobacteriaceae* | *Shigella/Escherichia* |  |
| *Proteobacteria* | *Gammaproteobacteria* | *Pseudomonadales* | *Pseudomonadaceae* | *Pseudomonas* |  |
|  |  |  |  |  |  |
| 8 probes detected bacteria at higher taxonomic levels: | | |  |  |  |
| *Actinobacteria* |  |  |  |  |  |
| *Firmicutes* |  |  |  |  | *Firmicutes* *Clostridia*, *Bacilla* |
| *Firmicutes* | *Clostridia* | *Clostridiales* | *Peptostreptococcaceae* |  | *Clostridium* *difficilie*/*Clostridiales* and *Veillonella* |
| *Firmicutes* | *Clostridia* | *Clostridiales* | *Lachnospiraceae* |  | *Lachnospiraceae* Incertae Sedis |
| *Firmicutes* |  |  |  |  | *Firmicutes* *Lactobacillales*, *Clostridium* *perfringens*., *Staphylococcus* |
| *Firmicutes* |  |  |  |  | *Megasphaera*/*Clostridiales* and *Veillonellaceae* |
| *Proteobacteria* |  |  |  |  |  |
| *Proteobacteria* | *Gammaproteobacteria* | *Enterobacterales* |  |  | *Salmonella, Citrobacter, Cronobacter, Enterobacter, Morganella* |
